# Age-Friendliness and Life Satisfaction of Young-Old and Old-Old in Hong Kong

**DOI:** 10.1155/2017/6215917

**Published:** 2017-02-24

**Authors:** Alma M. L. Au, Stephen C. Y. Chan, H. M. Yip, Jackie Y. C. Kwok, K. Y. Lai, K. M. Leung, Anita L. F. Lee, Daniel W. L. Lai, Teresa Tsien, Simon M. K. Lai

**Affiliations:** Department of Applied Social Sciences, The Hong Kong Polytechnic University, Hung Hom, Hong Kong

## Abstract

Age-friendliness, promoted by the World Health Organization (WHO), aims to enable and support individuals in different aspects of life for fostering life satisfaction and personal well-being as they age. We identified specific aspect(s) of age-friendliness associated with life satisfaction and examined similarities and differences in age-friendliness and life satisfaction in young-old and old-old adults. Six hundred and eighty-two ageing adults were asked to complete a survey questionnaire consisting of the Age-friendly City Scale, Satisfaction with Life Scale, and sociodemographic variables. Multiple linear regression analysis was used to examine the effects of various domains of age-friendliness on life satisfaction among the young-old adults (aged 65 to 74, *n* = 351) and the old-old adults (aged 75 to 97, *n* = 331). Common domains associated with life satisfaction in both young-old and old-old groups were transportation and social participation. Community and health services were associated with life satisfaction for the young-old group only. On the other hand, civic participation and employment was significantly associated with the old-old group only. Social participation is important for the young-old and the old-old. Ageing older adults can be a resource to the society. Implications for promoting and implementing age-friendliness were discussed in the context of successful and productive ageing and the need for a more refined taxonomy of social activities.

## 1. Introduction

As in other Asian countries, Hong Kong is encountering increasing population ageing. According to the Hong Kong Population Projections Report, the population of adults 65 and over will increase from 15% in 2014 to 31% in 2044 [1]. Hong Kong has the longest life expectancy in the world, and the average lifespan for both genders is around 84 years [2]. With the aged population boom and longer life expectancy, the Hong Kong Government has provided targeted services and subsidies. The age of 65 is the typical benchmark to define eligibility for most of the relevant welfare supports, including the Old Age Living Allowance [3]. Building an age-friendly environment is one of the major targets highlighted in the 2016 Policy Address of the Hong Kong Government [4]. In addition to addressing the financial burden of the ageing population in terms of retirement protection, medical care, and elderly services, the Policy Address underscored that the new generation of aged adults will be healthier, better educated, and fully capable of making further contributions to the community. Initiatives include enhancing barrier-free access, outdoor facilities, and providing a safe and comfortable home environment and digital inclusion for age individuals.

The age-friendly city concept is based on the framework for active ageing defined by the World Health Organization (WHO), rooted in the belief that a supportive and inclusive environment will enable residents to optimize health, participation, and well-being as they age successfully in the place in which they are living without the need to move [5–7]. The eight domains or features of age-friendly city encompass aspects ranging from physical infrastructure to social environment and include (i) outdoor spaces and buildings, (ii) transportation, (iii) housing, (iv) social participation, (v) respect and social inclusion, (vi) civic participation and employment, (vii) communication and information, and (viii) community and health services [8]. This framework has been applied in various countries to gauge the age-friendliness of retirement communities [9] and of transport and health services [10, 11]. According to a study conducted in two districts in Hong Kong, many age-friendly features exist but there is still substantial room for improvement in developing more comprehensive and specific long-term action and evaluation plans. The authors recommended that mechanisms be established to involve all aged adults, government officials, politicians, and service providers in conducting a baseline assessment and developing a three-year age-friendly action plan [12]. This study described in this paper is a part of this follow-up initiative [13].

The purpose of this study was to identify specific aspect(s) of age-friendliness associated with life satisfaction and examine similarities and differences in age-friendliness and life satisfaction in young-old and old-old adults. The concept of age-friendly city has based its formulation on active ageing [5–7]. Various definitions of active and successful ageing have been proposed. These concepts and models can be helpful in furthering our understanding of how age-friendly models can contribute to the well-being of aged adults. Active and successful ageing includes having a positive sense of oneself and relations with others, acceptance of the past and the present, autonomy and control over one's environment, participation in activities, and having a purpose in life [14]. More recent criteria have highlighted the need for testable components, including healthy lifestyle, maintaining high functioning levels, and active social engagement [5, 15]. Process-orientated models such as the selective optimization with compensation model depict the flexible adjustment of goals in relation to age-related losses [16]. The socioemotional selectivity theory underscores that the sense of limited future time motivates aged people to prioritize goals intended to derive emotional meaning from life [17]. The differentiation of process from outcomes enables the inclusion of age-graded expectations and the subjective criteria of psychological experience such as life satisfaction or psychological well-being [18].

Neugarten and colleagues considered age norms in regulating the life course [19]. These norms are culturally affected and act as a “social clock” to inform individuals of the appropriate time for a certain life event. Neugarten has also suggested that there might be differences in age norms even among aged adults [20, 21]. She identifies two major groups of aged adults: the “young-old,” aged 55 to 75, and the “old-old,” aged 75 or above. Different aged individuals are able to judge and set their corresponding social clocks. Satisfaction or distress may result based on the subjective evaluation of personal life.

With reference to successful or active ageing, there is a close association between subjective well-being and life satisfaction [22]. Life satisfaction, the cognitive component of well-being, refers to an individual's evaluation of their life [23]. A great number of aged adults have reported higher well-being linked to participation in social and leisure activities [24].

According to a study by Jeste et al. [25], despite physical and cognitive declines, older age was found to be associated with higher levels of resilience and lower levels of depression. Explanations put forward include contentedness with accomplishments in life and a more realistic appraisal of one's own strengths and limitations include after surviving various hardships. Resilience is often understood as response to acute stress. At the same time, adaptive self-appraisal may be an important aspect of maintaining well-being in the context of losses in functioning with ageing.

With the emergence of Neugarten's view of young-old and old-old, more researchers have focused on these categories. However, existing studies have focused more on the young-old, since they shared some common characteristics. For instance, they are entering their first retirement and are relatively healthy, better educated, and active in social and political participation. One study has shown higher levels of well-being associated with volunteering among young-old adults [26]. With respect to group differences, the young-old have been found to possess higher positive well-being and self-perceived successful ageing than the old-old [27, 28]. Concerning mental or physical health issues, the prevalence of depressive symptoms has been found to be highest in oldest-old adults compared to other aged groups [29]. Another study identified significant associations between depression and malnutrition in the young-old but not the old-old [30]. In a previous study of 254 aged adults, Au and her colleagues found that while life satisfaction was significantly related to the fulfilment of achievement and affiliation for the young-old, life satisfaction was significantly associated with the fulfilment of altruistic goals for the old-old [31].

While previous studies have examined differences in ageing experiences between age groups of aged adults, to our knowledge, no studies have examined the specific relationship between age-friendliness and life satisfaction with reference to the young-old and old-old. Neugarten's model can have important implications for the understanding of how age-friendly concepts can contribute to the life satisfaction of aged adults. Therefore, the aims of the present study include (1) to examine the relationship between specific sociodemographic variables and life satisfaction among young-old and old-old adults, (2) to assess the relationship between specific domains of age-friendliness and life satisfaction with controlled sociodemographic variables, and (3) to identify similarities and differences in specific domains of age-friendliness associated with life satisfaction between young-old and old-old adults.

## 2. Materials and Methods

### 2.1. Participants

Study participants were recruited mainly from community centres and nongovernmental organizations (NGOs) in Kowloon East Region including Kowloon City and Kwun Tong, using a convenience sampling approach. Inclusion criteria for participants included being Cantonese speakers, having comprehensive understanding without wearing a hearing aid, and being mentally sound. The original study aimed at exploring the age-friendliness of Hong Kong as perceived by individuals aged 18 or older. For the purpose of this study, we only select adults aged 65 and above for data analysis.

### 2.2. Measures

All participants were asked to complete a structured questionnaire, which consisted of three main parts: the Age-friendly City Scale, the Satisfaction with Life Scale, and basic demographic variables.

#### 2.2.1. The Age-Friendly City Scale (AFCS)

Based on the WHO age-friendly city guidelines, it was adopted and translated into Chinese [32]. Eight domains were assessed using 53 items. Participants were asked to rate their views on each item using a 6-point Likert-type scale ranging from 1 (“strongly disagree”) to 6 (“strongly agree”). Responses for each domain were averaged as a mean score. The reliability (Cronbach's alpha) estimate for each domain ranged from .66 to .81. Example items for each domain are included in Appendix.

#### 2.2.2. The Satisfaction with Life Scale (SWLS)

It was used to measure the cognitive component of subjective well-being [33]. It consisted of five items, such as “I am satisfied with my life.” Participants rated each item on a 7-point Likert-type scale ranging from 1 (“strongly disagree”) to 7 (“strongly agree”). The average score of this scale was used for analysis. The reliability of SWLS was .87 and over .90 in this study and our previous study, respectively [31].

#### 2.2.3. Sociodemographic Variables

Sociodemographic variables including age, gender, education level, health status, marital status, income, and expenditure were used in the analysis as factors to be controlled statistically. These variables have been consistent in predicting life satisfaction in previous research [34]. Gender was coded as 1 (“male”) or 2 (“female”). Participants aged 65 to 74 were categorized as “young-old” and those aged 75 or as “old-old.” Education level was measured in eight categories referring to the local education system, ranging from 1 (“never”) to 8 (“degree level or above”). Health condition and expenditure were measured using 5-point Likert-type scales, ranging from 1 (“bad”) to 5 (“excellent”) and from 1 (“very insufficient”) to 5 (“very sufficient”), respectively. Income was categorized into 14 options ranging from 1 (“less than $2000”) to 14 (“more than 100,000”). Although educational level and income were grouped as presented in Table 1, original values were used for data analyses.

### 2.3. Procedures

Participants were introduced the background of our study by research helpers, who were trained master students and research assistants in the field of Psychology as well as members from the Institute of Active Ageing. After signing the informed consent form, a set of questionnaires would be given. A few aged participants had difficulties in reading or writing, and research helpers assisted these participants to complete the questionnaire by reading out the questions and asking them to denote their response to each item. Participants were given a supermarket coupon after completing the questionnaire.

### 2.4. Statistical Analyses

Multiple linear regression analyses were performed using IBM SPSS Statistics 23. It tested the direction and strength of the association between predictor(s) and dependent variable(s). The predictors of SWLS were formed into two blocks and analysed hierarchically using the ENTER method. The first block of predictors includes sociodemographic variables and were evaluated as statistically controlled factors of SWLS. The second block of predictors included dimensions of AFCS and their predictive effects on SWLS were evaluated with the sociodemographic variables adjusted. 

## 3. Results

### 3.1. Descriptive Statistics

Differences in sociodemographic variables between young-old and old-old participants are shown in Table 1. The respective mean age of young-old and old-old participants was 68.91 (SD = 2.82) years and 80.29 (SD = 4.27), and most participants were women. Young-old participants reported a higher overall education level, with nearly 80% reporting primary or secondary education, while around 70% of old-old participants reported no education or primary education. In general, both young-old and old-old participants reported an adequate living standard, with around 15% reporting “bad” health status and around 15% reporting “insufficient” expenditure.

The mean AFSC domain scores of young-old and old-old participants are presented in Table 2. The scores for SWLS and six out of eight AFSC domains (outdoor spaces and buildings, transportation, housing, social participation, respect and social inclusion, and community, and health services) reported by old-old participants were significant higher than the scores reported by young-old participants (*p* < .05). There was no difference in scores for domains of civic participation and employment (*p* = .071) and communication and information (*p* = .091) between young-old and old-old participants. Both groups rated the social participation domain the highest and the community and health services domain lowest among the eight AFSC domains.

### 3.2. Association between Sociodemographic Variables and Life Satisfaction

The results of analyses for the first research question, the association between sociodemographic variables and life satisfaction, are presented in Tables 3 and 4. In both young-old and old-old groups, SWLS scores were significantly correlated (*p* < .05) with sociodemographic variables of expenditure (*r* = .36 and *r* = .34), income (*r* = .17 and *r* = .11), and health status (*r* = .24 and *r* = .39). Gender was significantly correlated with SWLS score only in the young-old group (*r* = −.12). The results of the regression analysis show that gender, marital status, educational level, income, and expenditure explained a significant amount of variance in SWLS score in both young-old (*R*^2^ = .18, *F*(6,344) = 12.17, *p* < .01) and old-old (*R*^2^ = .23, *F*(6,324) = 16.04, *p* < .01) groups. In both groups, expenditure (*β* = .31 and *β* = .25, *p* < .05) and health (*β* = .16 and *β* = .33, *p* < .05) had significant positive regression weights on SWLS score, indicating that those who perceived they had more than enough to spend and better health status would show better life satisfaction.

### 3.3. Association between Age-Friendly City Domains and Life Satisfaction

The results of analyses addressing the second research question, the association between AFCS and life satisfaction, are presented in Tables 5 and 6. SWLS scores were positively associated with scores on all AFCS domains. After controlling the effect of demographic variables, results of the regression analyses showed the association of AFCS scores with SWLS scores, with a statistically significant model for young-old (Δ*R*^2^ = .14, *F*(14,336) = 11.1, *p* < .01) and old-old (Δ*R*^2^ = .15, *F*(14,316) = 8.95, *p* < .01) groups. Similarities and differences in domains of age-friendliness associated with life satisfaction between young-old and old-old groups were then addressed within the models. The domain scores for transportation (*β* = .14 and *β* = .14, *p* < .05) and social participation (*β* = .21 and *β* = .15, *p* < .05) were significantly associated with SWLS scores in both young-old and old-old, respectively. In addition, the domain score for community and health services was significantly correlated with SWLS scores only in the young-old group (*β* = .19, *p* < .05), while the domain score for civic participation and employment was significantly associated with SWLS only in the old-old group (*β* = .12, *p* < .05). In other words, young-old adults tended to be more satisfied in an environment with good transportation, social participation, and community and health services, while old-old adults were more satisfied with an environment with good transportation, social participation, and civic participation and employment.

## 4. Discussion

In this study, old-old participants reported significantly higher scores in most of the age-friendly city domains as well as the Satisfaction with Life Scale, compared to young-old participants. These findings support the idea of “paradox of well-being,” in which older adults maintain high subjective well-being even when facing different challenges [35]. Old-old adults have been reported to be more capable of shifting motivation from pursuing information to achieving emotional satisfaction under time-constraints, as postulated by the socioemotional selectivity theory [36]. This motivational shift allows for changes in attitude, including a better evaluation and acceptance of individual strengths and limitations [37]. As aged adults have been reported to perceive their stressors as less severe when compared to younger generations [38], it would be a plausible interpretation for the higher rating of life satisfaction among old-old participants than young-old participants.

Variables such as economic status and perceived health situation were positively correlated with life satisfaction in both young-old and old-old groups. These results support the findings of previous studies [34, 39–41]. The standardized Beta weight for health among old-old participants was greater than among young-old participants, indicating that perceived good health may be a more vital determinant of life satisfaction in later life. Marital status and educational level did not show any correlation with life satisfaction in the study sample, and, with women reporting lower levels of life satisfaction, gender was found to correlate with life satisfaction in the young-old but not the old-old group. These findings are largely consistent with the findings of previous studies [42–44]. However, the specific reasons are not identified in the present study. Possible reasons include adoption of multiple caregiving roles [43].

The main purpose of this study was to investigate the association between age-friendliness and life satisfaction after controlling for sociodemographic variables. In both young-old and old-old groups, all age-friendly domains were correlated with life satisfaction, and most of the domains were correlated moderately. With respect to the regression model, both transportation and social participation were significant correlates of life satisfaction for both young-old and old-old groups, indicating that aged adults are more satisfied if these two domains are fulfilled. Mobility (the ability to move oneself, including walking or using transportation) [45] and social participation are essential components of successful ageing and have been associated with proximity to resources and recreational facilities, social support, and having a driving license [15, 46]. The Hong Kong Government has launched the Public Transport Fare Concession Scheme, intended to enable elderly people to travel on designated public transport modes and services at a concessionary fare of two dollars per trip [47]. Greater availability of choices and cheaper fees for transportation might allow elderly people to engage more actively in society. On the other hand, the domains of civic participation and employment were significantly associated with life satisfaction for the old-old group.

Social and civic participation also emerged as major themes from the data generated from the eight focus groups involving eight-five older adults in Kwun Tong and Kowloon City [48, 49]. Participants remarked that there were a variety of affordable activities that they could participate in community settings. However, more quota and more convenient locations were suggested. Apart from leisure activities, participants also highlighted a need for more productive activities like volunteering and even employment. Moreover, there was a need for more regular channels for older adults to express their opinions. Finally, more relief provisions were underscored to facilitate the participation of caregivers.

Making communities more age-friendly involves both physical and social infrastructure to enable older adults to participate in life-long activities in meaningful ways. Through personal and social engagement in the community, social inclusion is promoted and social capital is enhanced [50, 51]. This engagement could be achieved through contributions to paid and unpaid work, such as voluntary work [6]. Arguing that there can be more than one pathway through which social engagement can promote well-being, the ascribed value as well as purpose/goal or the meaning of the activities can be crucial [24]. Critical reviews of successful ageing have challenged the notion of promoting normative ideals for ageing in the context of social and civic engagement [52]. By implying the measurement of the value of a person based doing rather than being, we may fail to take into account the notion that there are structural inequalities in access to prescribed contributory roles for some marginalized groups due to differences in education, income, gender, social connections, health problems, and job opportunities [28, 53]. Thus, various crucial barriers are needed to be addressed before promoting social engagement for aged adults: profound involvement in caregiving, compulsory altruism, personal resources, and objective perceived and subjectively available engagement opportunities [54]. As such, an alternative model of ageing should be based on equal regard for all persons, taking into account of the diversity of aged adults and ageing processes as well as the oldest-old who are suffering from functional deterioration. These present findings have highlighted the importance of providing both young-old and old-old with an opportunity to contribute in the society leading to a sense of accomplishment and satisfaction [55, 56].

Community and health services were significantly associated with life satisfaction for the young-old group but not the old-old. These findings also echo previous findings in Hong Kong Chinese population. Cheng [57] measured aspiration–achievement discrepancies in three specific areas—material resources, social relationships, and health—in groups of older, middle-aged, and younger adults. Older adults had significantly smaller discrepancies in material resources and social relationships than the younger age groups but larger health discrepancies. However, though aspiration–achievement discrepancies in health increased with age, it had smaller effects on subjective well-being as compared to discrepancies in relationships. While some explanation may be provided in the context of the socioemotional selectivity theory [36], further investigations will be needed for ageing in the very old who may have adjusted to the health system.

The present study has also indicated suggestions for improvement in future research. Since the concept of age-friendly city was adopted from the WHO guidelines, some questions may not be pertinent in local context. For instance, cycle paths are not common facilities in all areas of Hong Kong. Moreover, volunteering may involve both social and civic participation [58]. Levasseur and her colleagues conducted a systematic review on how to define social participation in gerontology. They have suggested a common definition and a taxonomy of social activities linked with the ecological model [59]. This alternative conceptualization may suggest flexibility to integrate items in the domain of social participation and civic participation and employment for further investigation. Nevertheless, social and psychological functioning may change over time until very old age. For example, the association between social networks and social participation for middle-aged and elderly adults is confirmed in their longitudinal study [60]. A longitudinal design is advised to investigate the effect and changes at different time points so that clearer relationships between variables could be revealed. This is vital as the importance of social participation persists in the old-old.

## 5. Conclusion

Age-friendliness is significantly related to the life satisfaction of the ageing population. Social participation is important for the young-old and the old-old. Ageing older adults can be a resource to the society. The findings of this study have delineated some of the similarities and differences of needs between young-old and old-old adults. Future directions will need to work on a more specific taxonomy of possible social activities and social participation and how they can take place in the living environment of aged adults to enhance their well-being and their contribution to the younger generations.

## Figures and Tables

**Table 1:** Descriptive statistics of sociodemographic variables (mean and SD in age) by age group.

Variables		Young-old	Old-old
		(*n* = 365)	(*n* = 354)
Age	Mean ± SD	68.91 (2.82)	80.29 (4.27)

	Levels	Percentage	

Gender	Female	72.1%	65.8%
	Male	27.9%	34.2%

Marital status	Single	6.8%	2.3%
	Married	60.8%	44.9%
	Widowed	24.7%	47.7%
	Divorced	6.6%	4.3%
	Others	1.1%	0.8%

Education level	Never	12.6%	32.2%
	Primary school	39.6%	41.5%
	Secondary	39.8%	23.2%
	Diploma or higher diploma	2.8%	2%
	Degree or above	5.2%	1.1%

Health	Bad	15.1%	15.8%
	General	52.1%	50.0%
	Good	19.7%	22.1%
	Very good	10.4%	9%
	Excellent	2.7%	3.1%

Expenditure	Very insufficient	1.4%	1.7%
	Insufficient	14.5%	13.6%
	Just enough	64.1%	66.1%
	Sufficient	17.5%	16.9%
	Very sufficient	2.5%	1.7%

Income	<6000	54.8%	76.3%
	6001–10000	20%	11.8%
	10001–20000	11.5%	5.4%
	20001 or above	4.7%	2.5%
	N/A	9%	4%

**Table 2:** Mean scores of age-friendly city domains and satisfaction with Life Scale of young-old and old-old participants.

Domain	Young-old		Old-old		*p* value
	Mean	SD	Mean	SD	
Outdoor spaces and buildings	4.10	0.72	4.21	0.68	.049
Transportation	4.39	0.63	4.56	0.53	<.01
Housing	3.97	0.93	4.15	0.97	.013
Social participation	4.56	0.68	4.68	0.67	.024
Respect and social inclusion	4.22	0.78	4.34	0.78	.028
Civic participation and employment	4.01	0.88	4.13	0.95	.071
Communication and information	4.16	0.77	4.26	0.74	.091
Community and health services	3.85	0.78	4.10	0.69	<.01
SWLS	4.59	0.95	4.88	1.00	<.01

**Table 3:** Correlations and results from regression analyses of demographic variables in association with SWLS score in young-old participants.

Variables	Pearson correlation (*r*)						^B^ *β*
	^A^SWLS	Gender	Marriage	Education level	Expenditure	Income	
Gender	−.12^*∗*^						−.122^*∗*^
Marriage	−.06	.14^*∗*^					.011
Education level	.04	−.22^*∗*^	−.21^*∗*^				−.076
Expenditure	.36^*∗*^	.01	−.14^*∗*^	.16^*∗*^			.312^*∗*^
Income	.17^*∗*^	.04	−.09	.16^*∗*^	.30^*∗*^		.079
Health	.24^*∗*^	−.17^*∗*^	−.10^*∗*^	.17^*∗*^	.21^*∗*^	.11^*∗*^	.161^*∗*^

^*∗*^Statistically significant at *p* < .05 level.

*Note*. ^A^SWLS referred to the Satisfaction with Life Scale.

^B^Standardized coefficients beta of predictors of SWLS score are presented.

**Table 4:** Correlations and results from regression analyses of demographic variables in association with SWLS score in old-old participants.

Variables	Pearson correlation (*r*)						^B^ *β*
	^A^SWLS	Gender	Marriage	Education level	Expenditure	Income	
Gender	.07						.096
Marriage	.01	.26^*∗*^					.018
Education level	−.08	−.31^*∗*^	−.14^*∗*^				−.101
Expenditure	.34^*∗*^	−.06	−.08	.16^*∗*^			.253^*∗*^
Income	.11^*∗*^	.00	−.13^*∗*^	.13^*∗*^	.28^*∗*^		.009
Health	.39^*∗*^	−.13^*∗*^	−.08	.05	.32^*∗*^	.13^*∗*^	.328^*∗*^

^*∗*^Statistically significant at *p* < .05 level.

*Note*. ^A^SWLS referred to the Satisfaction with Life Scale.

^B^Standardized coefficients beta of predictors of SWLS score are presented.

**Table 5:** Correlations and results from regression analyses of AFC scores in association with SWLS score in young-old participants.

	Pearson Correlation (*r*)								^B^ *β*
	^A^SWLS	Outdoor spaces and buildings	Transportation	Housing	Social participation	Respect and social inclusion	Civic participation and employment	Communication and information	
Outdoor spaces and buildings	.23^*∗*^								−.030
Transportation	.29^*∗*^	.70^*∗*^							.141^*∗*^
Housing	.25^*∗*^	.42^*∗*^	.39^*∗*^						.088
Social participation	.30^*∗*^	.41^*∗*^	.45^*∗*^	.41^*∗*^					.207^*∗*^
Respect and social inclusion	.21^*∗*^	.53^*∗*^	.55^*∗*^	.37^*∗*^	.59^*∗*^				−.136
Civic participation and employment	.09^*∗*^	.34^*∗*^	.35^*∗*^	.27^*∗*^	.39^*∗*^	.49^*∗*^			−.090
Communication and information	.19^*∗*^	.46^*∗*^	.38^*∗*^	.38^*∗*^	.41^*∗*^	.56^*∗*^	.41^*∗*^		.050
Community and health services	.31^*∗*^	.51^*∗*^	.53^*∗*^	.42^*∗*^	.43^*∗*^	.57^*∗*^	.34^*∗*^	.49^*∗*^	.194^*∗*^

^*∗*^Statistically significant at *p* < .05 level.

*Note*. ^A^SWLS referred to the Satisfaction with Life Scale.

^B^Standardized coefficients beta of predictors of SWLS score are presented.

**Table 6:** Correlations and results from regression analyses of AFC score in association with SWLS score in old-old participants.

	Pearson correlation (*r*)								^B^ *β*
	^A^SWLS	Outdoor spaces and buildings	Transportation	Housing	Social participation	Respect and social inclusion	Civic participation and employment	Communication and information	
Outdoor spaces and buildings	.29^*∗*^								−.021
Transportation	.36^*∗*^	.63^*∗*^							.136^*∗*^
Housing	.33^*∗*^	.42^*∗*^	.41^*∗*^						.049
Social participation	.35^*∗*^	.43^*∗*^	.44^*∗*^	.47^*∗*^					.147^*∗*^
Respect and social inclusion	.30^*∗*^	.51^*∗*^	.48^*∗*^	.43^*∗*^	.51^*∗*^				.002
Civic participation and employment	.35^*∗*^	.38^*∗*^	.37^*∗*^	.31^*∗*^	.38^*∗*^	.47^*∗*^			.117^*∗*^
Communication and information	.27^*∗*^	.37^*∗*^	.34^*∗*^	.34^*∗*^	.44^*∗*^	.46^*∗*^	.38^*∗*^		.065
Community and health services	.34^*∗*^	.42^*∗*^	.50^*∗*^	.35^*∗*^	.40^*∗*^	.44^*∗*^	.42^*∗*^	.41^*∗*^	.060

^*∗*^Statistically significant at *p* < .05 level.

*Note*. ^A^SWLS referred to the Satisfaction with Life Scale.

^B^Standardized coefficients beta of predictors of SWLS score are presented.

**Table 7:** 

Domain	Sample item
Outdoor spaces and buildings	Public areas are clean and pleasant
Transportation	Public transportation costs are consistent, clearly displayed and affordable
Housing	Sufficient and affordable home maintenance and support services are available
Social participation	Activities and events can be attended alone or with a companion
Respect and social inclusion	Older people are regularly consulted by public, voluntary and commercial services on how to serve them better
Civic participation and employment	A range of flexible options for older volunteers is available, with training, recognition, guidance and compensation for personal costs
Communication and information	Regular information and broadcasts of interest to older people are offered
Community and health services	Home care services include health and personal care and housekeeping

## Appendix

See Table 7.
